# A Systems Approach to Performance Analysis in Women’s Netball: Using Work Domain Analysis to Model Elite Netball Performance

**DOI:** 10.3389/fpsyg.2019.00201

**Published:** 2019-02-06

**Authors:** Scott Mclean, Adam Hulme, Mitchell Mooney, Gemma J. M. Read, Anthony Bedford, Paul M. Salmon

**Affiliations:** ^1^Centre for Human Factors and Sociotechnical Systems, University of the Sunshine Coast, Sippy Downs, QLD, Australia; ^2^Department of Movement Science, Australian Institute of Sport, Canberra, ACT, Australia; ^3^School of Exercise Science, Australian Catholic University, Melbourne, VIC, Australia; ^4^School of Health and Sports Sciences, University of the Sunshine Coast, Sippy Downs, QLD, Australia

**Keywords:** netball, performance analysis, work domain analysis, coaching, women’s sport

## Abstract

Netball is a newly professional women’s sport, as such there has been little research conducted investigating performance analysis (PA) in elite netball. The aim of this study was to develop a model of the elite netball performance system to identify the complex relationships among key performance indicators. Eleven elite subject matter experts (SMEs) participated in workshops to produce a systems model of the netball match performance. The model was developed using the work domain analysis (WDA) method. A model of the netball match performance system was produced showing the interrelated objects, processes, functions, values, and purposes involved in elite level netball matches. The model identified the components of elite level netball performance and the interactions and relationships between them. The output of this research has identified novel PA measures including passing and possession measures, measures of cognitive performance, and measures related to physical activity. Netball is a complex sport, involving multiple dynamic and interrelated components. Consequently, there is an opportunity to develop holistic PA measures that focus on interacting components, as opposed to components in isolation.

## Introduction

Netball is one of the most popular women’s sports, and is played by more than 20 million people across more than 80 countries (Netball Australia, 2018). Since 1963 a netball World Championship tournament has been played every 4 years. In 1990 netball first featured in the Commonwealth Games as a demonstration sport, and has since been played competitively for medals since 1998 (Netball Australia, 2018). Despite substantial international popularity, netball remained an amateur sport for several decades. However, netball now has 61 countries recognized by the International Netball Federation, which includes several professional netball leagues. This recently obtained professional status of netball will no doubt generate new pressures and demands common within professional sport, one of which will be the increased demand for successful on-court match performance. To optimize match performance, it is necessary to assess performance variables in order to provide practical feedback to players and coaches, which will guide coaches decision making and subsequently the coaching process (Bishop, 2008).

Performance analysis (PA) in sport involves analysing and evaluating the technical, tactical, physical, and cognitive aspects of performance in training and competition to better understand the components of successful performance (Hughes and Franks, 2004; Bishop, 2008). There is a substantial body of PA research associated with long standing professional sports such as football (Sarmento et al., 2014, 2017), basketball (Bourbousson et al., 2010; Gómez et al., 2013; Sampaio et al., 2015), handball (Meletakos et al., 2011; Oliveira et al., 2012; Lago-Peñas et al., 2013), rugby (Vaz et al., 2010; Bishop and Barnes, 2013; Higham et al., 2014), water polo (Gómez et al., 2014; Lupo et al., 2014; Ruano et al., 2016), and Australian rules football (Robertson et al., 2015, 2016). In netball, research has focused on understanding the players’ physical (Davidson and Trewartha, 2008; Cormack et al., 2014; Bailey et al., 2017; Thomas et al., 2017), biomechanical (Fish and Greig, 2014; Sinclair et al., 2015), and anthropometric (Hopper et al., 1995) characteristics in isolation, rates of injury occurrence, and injury prevention strategies (Hume and Steele, 2000; McManus et al., 2006; Mason-Mackay et al., 2016). Although these studies have been important for understanding the physical demands of netball and have demonstrated the differences in variables between different levels of competition, they do little to describe on-court performance. As such, there is a paucity of PA research in netball that has attempted to integrate multiple components of match performance, or to understand team functioning. However, recent research has demonstrated that a complex system approach is necessary to understand sports performance, which requires a more holistic approach than isolated measurement of performance variables (McLean et al., 2017b; Salmon et al., 2017a; Hulme et al., 2018).

Previous research on the technical and tactical components of PA in netball has typically used descriptive notational analysis (frequencies and percentages) of isolated performance variables for individual players such as passing, turnovers, and shooting to determine successful netball performance (O’Donoghue et al., 2008; Pulling et al., 2016). This suggests that the current understanding of the composition of successful netball performance, and the appropriate methods to measure them is limited (Croft et al., 2017). A limitation of this approach in netball, and generally for team sports, is that components of performance are often investigated in isolation without consideration of the interactions between other components of performance (Rein and Memmert, 2016; McLean et al., 2017b). This represents a deterministic and reductionist approach to PA and does not consider interdependencies or the relational perspectives within and between teams that is required to understand and describe complex team performance (Mackenzie and Cushion, 2013; McLean et al., 2017b).

In sports PA research, it is becoming increasingly accepted that measuring isolated PA variables without considering the interactions between them, and the influence of match context, is limited and cannot appropriately explain team sports performance (McLean et al., 2017b; Sarmento et al., 2017). In addition, such analyses contribute to a research-practice gap, whereby the reductionist analyses are of limited use to coaches for the design of training practices (Bishop, 2008; McLean et al., 2017b). Invasion sports such as netball represent complex systems, where multiple human and non-human components are operating within a dynamic and constantly changing match environment (Duarte et al., 2012, 2013; McLean et al., 2017b). Investigating sporting performance as complex systems allows an understanding of the cooperative behaviors of team members in space and time, as well as emergent behaviors based on the opposition actions (Balague et al., 2013; Travassos et al., 2013). Traditional sport science research applications that measure individual performance variables cannot provide a complete understanding of the complexity of performance or the factors influencing it, nor do they allow the detection of new emergent behaviors that could improve performance, or how performance can be measured (Carling et al., 2014). Recent studies have demonstrated the benefit of analysing and understanding sporting performance using methods specifically designed for analysing complex systems (McLean et al., 2017b; Salmon et al., 2017a; Hulme et al., 2018). Systems ergonomics methods are specifically designed to understand complex system behavior, for example, one study used cognitive work analysis (CWA), a systems ergonomics method, to model the football performance system to identify the components of football performance, and the interactions between them (McLean et al., 2017b). Importantly, the use of subject matter experts (SMEs) in the development of the football performance model helped to provide practical insights for coaches that are both important to measure that are representative of the state of the system, and those that have a limited use for coaches (McLean et al., 2017b). In addition, over the past 5 years, there has been a progressive increase in team analysis measures based on positional data (Bartlett et al., 2012; Frencken et al., 2012; Moura et al., 2012), and teamwork interactions based on network analysis, in football and basketball (Clemente et al., 2015a; Gonçalves et al., 2017; McLean et al., 2017c). Moreover, at an elite level, coaches prefer emergent team PA measures compared to individual player analyses (McLean et al., 2017b). As such, the use of analysis methods to investigate group behaviors have improved our understanding of the complex and dynamic nature of team sports, and have provided usable information for coaches regarding team functioning (Sarmento et al., 2017).

The infancy of professional netball, and the subsequent lack of integrated PA research in elite level netball provides a unique opportunity to obtain a more complete understanding of the composition of performance in elite netball, to understand the interactions between components, emergent properties (Croft et al., 2017), and to develop a conceptual base for future research and PA in netball. By building on established PA research in other areas, it will be possible to optimize PA in netball, potentially skipping decades of research describing isolated and independent performance components, as has been the case in other sports (Mackenzie and Cushion, 2013). Therefore, the primary aim of the current study is to describe and model the netball performance system via the application of CWA, a systems ergonomic method that has been used to understand complex systems, and in the design and re-design of complex systems across multiple domains (see “Materials and Methods” section). This first-of-its-kind analysis will allow the identification of the different components of performance and how they interact to influence performance in netball. In turn, the model will support identification of appropriate PA measures for netball.

## Materials and Methods

### Participants

Ethical approval for this study was granted by the Human Research Ethics Committee at the University of the Sunshine Coast (A/17/1043). Eleven elite level SMEs participated in structured workshops to develop a systems analysis model of the netball match performance system. The SMEs were experienced elite level netball coaches (C), professional netball players (PL), netball performance analysts (PA), an exercise physiologist (EP), a sports psychologist (SP), a skill acquisition specialist (SA), a high-performance manager of a professional netball team (HPM), and a strength and conditioning coach (S&C) (Table 1).

**Table 1:** Subject matter expert (SME) characteristics.

SME	Professional experience (years)	Professional games played	Professional games coached	International games played	International tournaments	Number of other sports worked in	National sport academy (years)	Accreditation
C	18	∼350	∼250	42	–	–	–	–
C	12	∼50	76	–	–	–	8	HPC
PL	7	32	–	63	2	–	–	–
PL	3	35	–	10^∗^	1^∗^	–	–	–
PA	15	–	–	–	3	4	–	Ph.D.; ISPAS (L5)
PA	10	–	–	–	5	4	5	Ph.D.; AspS2; ISPAS (L5)
SP	22	–	–	–	–	7	–	AHPRA
EP	23	–	–	–	–	–	–	Ph.D.
S&C	2	–	–	–	2	3	–	ASCA (L2)
HPM	20	–	–	–	2	17	20	ASCA (L3)
SA	14	–	–	–	–	12	10	Ph.D.
Total	146	67	250	115	15	36	43	
Mean	13.3	33.5		38.3	2.5	9	10.8	

*^∗^World youth cup, ^∼^self-reported estimates. ‘Professional games coached’ includes assistant coach. ISPAS, International Society of Performance Analysis of Sport; ASCA, Australian Strength and Conditioning Association; AHPRA, Australian Health Practitioners Registration Agency; Ph.D., Doctor of Philosophy; HPC, High Performance Coach; ASpS2, Accredited Sports Scientist Level 2 – Exercise and Sports Science Australia. International tournaments include: Olympic Games, World Championships, Commonwealth Games, South Asian Games, World Youth Cups, Asian Champions Trophy.*

### Study Design

In the current study, WDA, the first phase of a systems ergonomics method, CWA, was used to develop a model of the netball match performance system to describe in detail the composition of netball performance (Naikar et al., 1999). CWA (Vicente, 1999) is a systems analysis and design framework that has previously been used in sport to analyze the football match performance system (McLean et al., 2017b) and other complex sociotechnical systems (Read et al., 2016; Salmon et al., 2016, 2017b), and to inform system design or redesign activities (Cornelissen et al., 2014; Stanton and Bessell, 2014). The framework comprises five phases; however, the phases applied is dependent on the purpose of the analysis. This study uses the first phase of CWA (Vicente, 1999), WDA. WDA is used to provide an event and actor independent model of the system under analysis: in this case the elite netball match performance system. The aim is to understand the constraints imposed on the actions of any player performing activities within a netball match environment. This is achieved by describing systems at five conceptual levels using the abstraction hierarchy (AH) method (McLean et al., 2017b) (Table 2).

**Table 2:** Abstraction hierarchy descriptions.

Abstraction hierarchy level	Question
1. Functional purposes	What is the reason for playing?
2. Values and priority measures	How can players, coaches assess whether the functional purposes are being achieved?
3. Purpose-related functions	What functions must be performed to achieve the purposes of the netball match system?
4. Object-related processes	What processes or affordances are provided by the physical objects in the netball match system?
5. Physical objects	What physical objects are in the netball match system?

**FIGURE 1:**
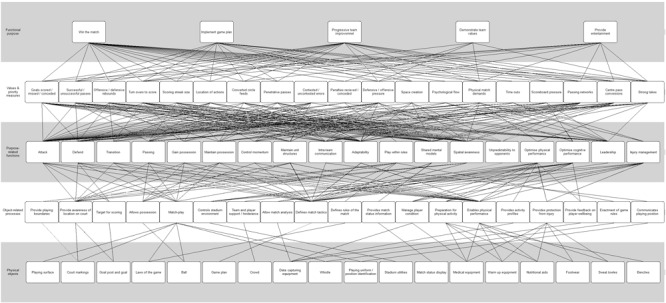
The work domain analysis of the netball performance system.

Within the WDA model the specific functional purposes, measures, functions, processes and physical objects are displayed as nodes. The nodes are linked across the AH levels via means-ends relationships (indicated by connecting lines in Figure 1). This indicates that linked nodes above a node in the hierarchy relate to why the content of the node is required, and the linked nodes below a node relate to how the node is achieved (Salmon et al., 2017b). For example, in the netball model the function ‘Attack’ might have links to values and priorities such as ‘Number of goals scored’ and ‘Scoring streak’ above, and links from object-related processes such as ‘Target for scoring’ and ‘Physical performance’ below.

### Procedure

The procedure for conducting a WDA requires an accepted nine step methodology (Naikar, 2013). The following section outlines the steps followed during the development of the current WDA.

#### Step 1: Establish Aims and Purpose

The purpose of the analysis was to develop a systems model of netball match performance, including a description of on-court performance, current measures used to assess performance, and to identify novel measures of performance.

#### Step 2: Anticipate Project Constraints

The main constraint considered was limited access to elite level SMEs due to the high demands placed on their time during training and competition. To manage this, we used multiple SMEs, including players, coaches, match analysts, and strength and conditioning coaches and held three separate workshops to provide as much opportunity for participation as possible.

#### Step 3: Define the System Boundary

To develop the WDA, it was necessary to set analysis boundaries. The boundary for the current analysis was confined to professional women’s netball match-play. Factors related to performance that occur outside of the match, such as training, nutrition, sleep, etc., were not considered in the analysis. A second aspect of the analysis boundary was a specific focus on elite level women’s netball.

#### Step 4: Classify System Constraints

For the purpose of this study, the WDA constraints represent the specific types of relationships that are to be modeled between the different components of netball match performance. For example, the physical context in which netball players operate imposes constraints on the actions of individual and group behaviors, whether this be via a need to adapt to the opposing team or the pressure to perform under stressful passages of play. Given that the relationships among performance-based components are largely non-deterministic (i.e., players continuously exhibit large degrees of freedom and latitude for behavior), the type of data sources and development of the analysis needed to include the firsthand experience of ‘real’ people who could reflect on why certain actions and behaviors occur during on court performances.

#### Step 5: Locate Data Sources

Existing scientific research in elite-level netball and wider sports performance peer-reviewed academic literature, as well as the identified SMEs were considered appropriate data sources to develop an initial WDA. In addition, the first author attended multiple training sessions of a professional netball team prior to the analysis to assist in understanding what comprises netball performance. The knowledge gained enabled recommendations of PA measures from other sports that could be modified and used in netball to assess performance.

#### Step 6: Construct the WDA

An initial model of the netball match system was developed by the research team based on the data collected in step five. The research team comprised four researchers experienced in applying systems ergonomics methods to sporting domains (Hulme et al., 2017; McLean et al., 2017b; Salmon et al., 2017a), two of which are human factors (HFs) practitioners with extensive experience in applying CWA in systems analysis and design across a range of safety critical domains (Read et al., 2015; Salmon et al., 2016), and two experienced netball performance analysts. Due to the scarcity of netball specific PA measures, several of the nodes within the initial WDA were derived from the PA literature on well-established professional sports. The research team applied the means-ends-links to the final model, to show the connections between the nodes across the levels of the AH using the how-what-why prompts.

#### Step 7: Refine the Analysis

The draft WDA model was reviewed and refined by the SMEs in three separate workshops. The SMEs were questioned on the appropriateness of the nodes in the draft model, including whether each node should be included or excluded from the model, and if the terms used were applicable to netball practitioners and coaches. Where required nodes and relationships were either removed, modified, or added.

#### Steps 8 and 9: Review and Validate the WDA

The completed WDA was presented to the two expert netball performance analysts for review. They were asked to review each node within the model, starting at the functional purposes level and proceeding downward to the physical objects level. The analysts agreed with the nodes at each level and provided no further modifications to the WDA model. To determine the novelty of included PA measures, the first author reviewed the existing literature on PA in netball, and items that have not previously been assessed in netball literature were considered to be novel to netball PA.

## Results

### Modifications to the WDA

Based on the feedback from the SMEs during the workshops, there were modifications to the original WDA model of the netball performance system that was developed by the research team. See Appendix 1 for a summary of the SME modifications to the WDA model.

### Work Domain Analysis

The ‘netball match performance model’ is presented in Figure 1.

#### Functional Purposes

Five functional purposes identified highlighting the importance of winning the match, continual team improvement, adherence to the game plans, the demonstration of team values, and a need to entertain audiences.

#### Values and Priority Measures

Nineteen values and priority measures were identified. These included measures of goal scoring and passing, defense, cognitive measures of psychological flow and scoreboard pressure, team structure measures of space creation, and the strategic use of time outs.

#### Purpose Related Functions

Eighteen general functions were deemed necessary for achieving the functional purposes. These included match phase measures such as such as attack, defend, and transition. Functions of cognitive and physical performance, and tactical measures including controlling momentum, maintenance of structures, and adaptability.

#### Object Related Processes and Physical Objects

The two lower levels of the WDA show the physical objects in the system and the processes that each object supports in order to achieve the purpose-related functions. For example, the ‘Goal post and goal’ provides a ‘Target for scoring,’ and ‘Footwear’ provides ‘Protection from injury.’

#### Means-End-Links

**FIGURE 2:**
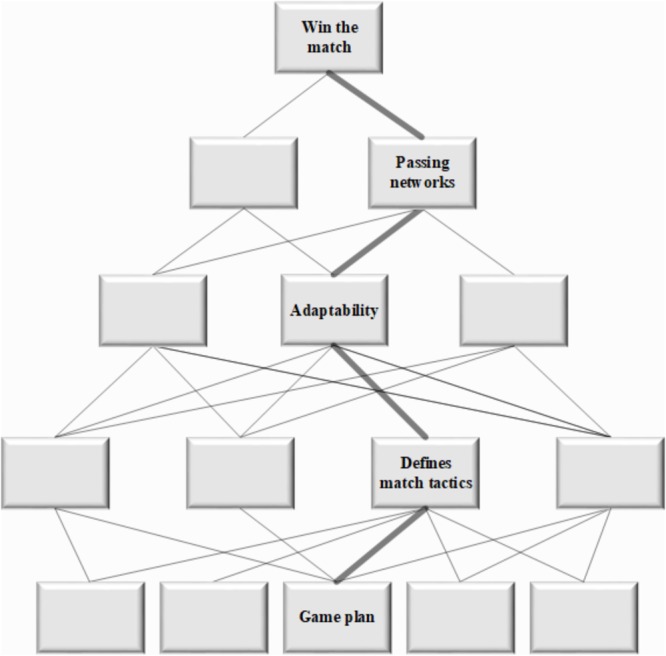
Example means-end-links pathway. The relationship between levels of the abstraction hierarchy are indicated by the means-end-links using a how-what-why triad. Working from bottom to top, the physical object ‘Game plan,’ enables the object related process of ‘Define match tactics,’ which includes the purpose related function of ‘Adaptability’ of which can be assessed by the ‘Passing networks’ which can be measured to determine its influence on the functional purpose ‘Win the match.’ This approach is completed for all components in the developed netball performance system model.

The means-end links in the WDA provide an indication of the relationships between the nodes across the five levels in terms of a how-what-why triad (Figure 2). The many-to-many means-end-links demonstrate the numerous possibilities for action available to the actors within the system (Figure 1).

## Discussion

The primary aim of this study was to develop, using WDA, a first-of-its-kind model of the netball performance system to determine the composition and interactions between the components of netball performance. Furthermore, the study aimed to identify components of performance that are not currently measured that have potential to advance PA in netball.

### Issues Identified With Current PA in Netball

The discussions with SMEs in the workshops, and information from the relevant netball PA literature, indicates that PA in elite level netball primarily uses isolated notational analysis measures of individual players to describe performance. For example, commonly used measures identified included successful and unsuccessful passes, offensive and defensive rebounds, and number of penalties received. This reductionist approach is further problematic as the SMEs identified a lack of integration of these measures. It is our contention that PA in netball can be enhanced by addressing these issues by incorporating measures to analyze team behaviors. These measures can be extracted from the WDA. The following sections will discuss the results from across the levels of the WDA, with a specific focus on the functions and measures novel to PA in netball. The discussed functions and measures will highlight potential analysis methods aimed at increasing the quality of feedback to coaches, and subsequently improve coach’s decision making to improve the coaching process. The measures and functions presented in the following sections are those that are deemed to be novel to netball, based on the current literature and from the workshop discussions with the SMEs. The two lower levels; object related processes and the physical objects from the WDA are not discussed further as they do not represent specific measures and functions of performance, which is the main focus of the study. They do, however, influence the behavior of the system which is indicated by the means-end-links (Figure 2).

### Functional Purposes

Five functional purposes were identified in the WDA (Figure 1). Although winning the match would logically be considered as the most important functional purpose, other functional purposes were identified, which were seen as changing in priority depending upon contextual factors. For example, the coaching staff and players may still be satisfied when losing if the team has implemented the game plan, progressively improved from previous performances, and demonstrated team values. The means-end links in the WDA demonstrate the interactions between the measures and functions, and the core functional purposes. However, it is questionable as to how appropriately the functional purposes within the system are currently measured, due to the reductionist tendencies of the notational analysis measures typically used for PA in netball. Using performance measures that cannot comprehensively measure the functional purposes identified in the WDA indicates that at present coaches may be required to subjectively assess the functional purposes of a match. This is potentially problematic as coaches use match performance statistics to reinforce their opinions of match events for decisions relating to future matches (Hughes and Bartlett, 2002). As such, basing decisions on variables that cannot fully capture the interactions of the components of performance, may lead to incorrect or misinformed decision making by coaches. In other words, “Most of us only see what we expect to see, and what we expect to see is what we are conditioned to see when we have learned the definitions and classifications of our culture” (Turner et al., 1967). Therefore, to optimize PA in netball, more sophisticated measures used in other more established professional sports (that are presented in the WDA) could be modified and used to objectively describe if the functional purposes are being achieved. In addition, the SMEs emphasized that as a relatively new professional women’s sport, it is important that netball matches provide entertainment in order to develop a strong supporter base, to gain sponsors, and to compete with other women’s sports to ensure long-term sustainability.

### Values and Priority Measures

Several of the values and priority measures from the WDA, including passing and goal scoring variables, turnovers, and rebounds have previously been measured to assess netball performance (O’Donoghue et al., 2008; Pulling et al., 2016; Croft et al., 2017). Although these match actions are important for successful performance in netball, they are often measured in isolation and without context, which limits their use for coaches (McLean et al., 2017b). Therefore, the model was used to identify novel measures of performances including group behaviors, measures from other sports that could be modified and applied to netball, outcome measures related to physical performance, and measures to optimize cognitive performance.

### Passing, Possession, and Shooting

**FIGURE 3:**
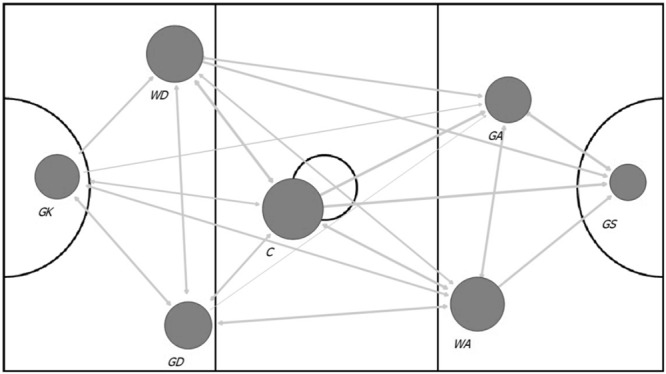
Network analysis diagram of passing in a netball match. The direction of attack is from left to right. In this conceptual example, the node (player) size is based on degree centrality (number of outward passes in the match). The larger nodes represent more passes made, and the edges (passes) are weighted by frequency of passes completed between players, e.g., thicker lines indicate a greater frequency of passes. GK (goalkeeper), GD (goal defense), WD (wing defense), C (center), WA (wing attack), GA (goal attack), GS (goal shooter).

Research on passing in netball has previously used descriptive notational analysis methods to determine performance (Pulling et al., 2016). Using isolated passing success or frequency to assess performance has decreased in many other sports and been replaced with more sophisticated methods (Gonçalves et al., 2017; McLean et al., 2017c, 2018b; Ribeiro et al., 2017; Mclean and Salmon, 2019). Therefore, to enhance PA in netball, the WDA included passing metrics used in other team sports to assess performance which have potential to be used for PA in netball. One of the aims of invasion team sports is to score points by overcoming the opposition through intra team coordination and passing (Eccles and Tenenbaum, 2004). Therefore, for the passing analyses to be effective, the methods applied need to explain this team connectivity and coordination. Network analysis is a commonly used method to assess passing interactions in other sports (Ribeiro et al., 2017). Network analysis is used to analyze passing networks and provides quantifiable metrics of the connectivity of the team, and identifies the influential players within the team (Clemente et al., 2015a,b; McLean et al., 2017a,c, 2018a). The advantage of measuring passing networks over traditional approaches is the consideration of interdependencies and relational perspectives relative to the entire team, compared to individual assessment of passing (Figure 3) (Wäsche et al., 2017). This provides information to coaches regarding team functioning, and potentially assesses whether match tactics are being achieved, or require changing. Therefore, the use of passing networks rather than isolated passing metrics (e.g., successful and unsuccessful passes) will provide additional information on team functioning in netball. In addition, the passing network analyses could be further enhanced when integrated with the location on the court where the passing networks commence, progress through the court, and break down (McLean et al., 2017c). This allows the identification of the areas of the court where passing networks were successful, the vectors of the passing network, or potentially where the opposition focuses their passing networks and their vectors (McLean et al., 2017c).

Passing metrics that identify and describe the penetration through opposition defenses, and spatial dominance are beginning to be used in other sports as a measure to assess the quality of passes (McLean et al., 2017b; Rein et al., 2017; Tenga et al., 2017). These metrics consider the number of opposition players bypassed by a single pass, which is an effective pass compared to a sideways or backward pass that enables the defenders to still be involved in that specific moment of the game. Given the use of these measure in other team sports, penetrative passes would be a useful measure in netball, which prompted its inclusion in the WDA. Although this approach represents a somewhat isolated passing metric, it is a good example of how isolated passing analysis can be used alongside contextual details to provide a detailed analysis of the effectiveness of a pass.

The analysis indicated that isolated types of passing that are currently measured, such as center passes, and goal circle edge feeds, should be reported with a shooting outcome. For example, instead of simply counting the center passes, and goal circle edge feeds, measuring the center passes that were converted, or the goal circle edge feeds that were converted would provide a more detailed analysis, and could be incorporated into the passing network analysis.

### Physical Performance Measures

Measuring physical performance in isolation, such as distance covered or the number of accelerations performed, also has limitations in relation to PA in sport, as physical outputs alone are not good indicators of overall performance (McLean et al., 2017b). Netball performance requires repeat explosive actions (Cormack et al., 2014), however, simply measuring the accelerations does not provide any information for how the accelerations benefit performance. In the model, strong takes were identified as a potential measure that has a performance outcome related to physical performance. Strong takes was defined as a received pass preceded by a rapid acceleration. Furthermore, acceleration data derived from motion tracking micro-technology could be a useful measure to determine effective defensive pressure, as it has been shown that percentage of passing occurrences is less in high compared to low defensive pressure for expert netball players (Bruce et al., 2009). Therefore, a measurement that describes the speed to apply defensive pressure, based on accelerations of the players may be a potential future measure when analysing performance in netball. Understanding physical match demands is important, especially from a load and injury prevention perspective (Bailey et al., 2017), however, more work is needed to integrate physical measures to performance outcome measures. Furthermore, the PA measures incorporating physical demands proposed here are somewhat subjective, suggesting that more sophisticated and objective methods are required.

### Optimizing Cognitive Performance

The SMEs indicated that based on previous experience psychological flow was identified as a key component of performance in elite level netball. Psychological flow in sport is explained as the optimal psychological state for performance (Csikszentmihalyi, 1990). Experiencing flow for an athlete is characterized by an intense focus and absorption in an activity, at the exclusion of all other thoughts and emotions (Swann et al., 2012). As a result, players acquire a sense of ‘everything coming together’ even in the most demanding situations (Swann et al., 2012). Interestingly, psychological flow in netball is not represented in the literature and the SMEs indicated that flow is not routinely assessed in professional netball. The absence of such measures are despite the existence of flow state scales developed to measure flow (Jackson and Marsh, 1996). Understanding the mechanisms of flow, and the behaviors and actions of players and teams demonstrated during flow states could enhance performance in netball. Importantly, as players share common experiences such as the same opponent, the same coach, and the same conditions of play, flow can be experienced at a team level (Bakker et al., 2011).

Minimizing scoreboard pressure errors was included in the WDA as an area where players’ cognitive performance could be enhanced. The effect of scoreboard pressure on the players, which typically occurs toward the end of quarters, was described by the SMEs as a critical phase in the match that can have a direct influence on netball performance and match outcome. For example, toward the end of matches and in overtime of close matches, basketball players may be overstimulated (anxiety and self-doubt) and therefore vulnerable to distractions (Hill et al., 2009, 2010; Gómez et al., 2015). This results in poorer decision making and subsequently turnovers of possession, compared to an optimally stimulated state (Hill et al., 2009, 2010; Bradley et al., 2011). Although, no research exits on scoreboard pressure in netball, coaches could improve this component of performance in training using time and match status constraints to replicate these periods of a match.

### Purpose Related Functions

The functions identified at this level included the high-level functions of attack, defend, gain and maintain possession, and components of these functions have been previously measured using a variety of methods (O’Donoghue et al., 2008; Pulling et al., 2016). However, several novel functions of netball performance were identified in the current model and require further investigation to optimize netball performance. These include controlling momentum, spatial awareness, unpredictability to opponents, and maintaining unit structures. Importantly, these functions consider the relational perspective of team members, and reduce the isolated type of analysis used so far in PA in netball research.

### Unpredictability to Opponents

Unpredictability to opponents in attack was identified and included as a function of performance in the WDA. Commonly termed entropy in sport science, unpredictability has been measured in other sports to determine the predictability of ball movements during play (Hobbs et al., 2018). In basketball, there is a relationship between teams who display entropy in offensive phases and the probability of a shot for an undefended player (D’Amour et al., 2015). A separate study investigating entropy of ball movements in basketball showed that in matches won, the team displayed increased entropy in the front court (scoring zone) compared to matches lost (Hobbs et al., 2018). This result not only shows the importance of being unpredictable in and around the scoring areas, but also the importance of analysing unpredictability in relation to elements of match context such as the location of actions. Given the use of entropy as measure of performance in basketball, it is logical that entropy could be a useful measure in netball to assess performance due to the similarities of the sports. In addition, this could be optimized in netball to include the entropy of the players spatial movements (Sampaio and Maçãs, 2012) in combination with the ball trajectories (Hobbs et al., 2018). An interesting future direction would be to investigate entropy as a function of match status, to assess differences in entropy whilst winning and losing netball matches.

### Maintenance of Unit Structures

The maintenance of team structures in sport is an integral component of successful performance (McLean et al., 2017b). As such, maintaining unit structure in netball is a critical strategy to (a) be compact defensively, and (b) destabilize the opposing teams’ defensive structures by increasing the distance between the opposition defenders when attacking (Moura et al., 2012). In football, during attacking phases of a match the players are more dispersed across the length and width of the pitch compared to defending situations where they are more compact (Moura et al., 2012). As an attacking team, an aim is to increase the dispersion of the defenders to attempt to create space for a shot at goal. For example, when the distance between defensive players in football is increased, more attempts at goal are conceded compared to when the distance between defenders is decreased (Moura et al., 2012). Although there is no research of this type in netball, research into team structures in other sports has shown the value of such information to understand the necessary components of creating attempts at goal. A difference between netball and other sports is that the match constraints in netball allow players of only certain positions to attack or defend. For example, only four players from each team can occupy the defensive zone, and only four players from each team can occupy the attacking zone of the court. For this reason, maintaining attacking or defensive units was included in the WDA rather than maintaining the entire team structure. There are various existing methods of assessing the maintenance of team structure, including stretch index, measurements of surface area, playing area, effective playing area, and centroid measures (Sarmento et al., 2017), all of which have potential to determine the maintenance of unit structures in elite netball.

### Controlling Momentum

The ability to control momentum in netball was supported in this analysis. The SMEs described controlling momentum in netball as the ability to slow down or speed up play as the match situation demands. Synonymous with controlling momentum is match tempo, which has not been clearly defined in sport science research (McLean et al., 2017b). There are conflicting definitions of tempo, and attempts to define it have included time in possession, speed of the ball, and speed of the players (Tenga et al., 2009; Buchheit et al., 2010; Wallace and Norton, 2014). However, a comprehensive definition of tempo, and the relationship between tempo and performance in sport is yet to be established (McLean et al., 2017b). The SMEs indicated that understanding the mechanisms of controlling momentum in netball would be beneficial to performance. Firstly, an appropriate method to determine how to measure tempo in netball is required, followed by methods to understand the player behaviors required to control it. This represents an opportunity for future research, and such results that would be informative and useable for coaches.

This first-of-its-kind WDA of the elite women’s netball performance system has demonstrated that netball is a complex system, which consists of multiple interacting and competing components of performance. The purpose of the WDA is not to determine the relative importance of individual nodes, but to capture all of the possibilities that influence performance. One of the strengths of WDA is that it does not attempt to describe or predict the behavior of the individuals within the analysis, but instead describes the constraints in the system that can influence and effect behavior (McLean et al., 2017b). This is important because coaches each have their own philosophies and preferred coaching styles. In the current study, it was important to model the existing measures of PA in netball, as well as to include novel functions of performance and potential measures that would exist in the optimal netball performance system. It is noted that the necessary resources required for measuring some of the proposed performance variables from the current study may not be readily available for many netball performance analysts at this early point in professional netball. This should represent a challenge to researchers to develop methods that are sophisticated and inexpensive, yet useable in practice. For instance, valid and reliable observational instruments for the technical and tactical analysis of rugby performance have been designed and could be extended to many of the novel netball performance measures proposed in this study (Villarejo et al., 2014). It has been shown here that by understanding the PA variables of other sports more advanced in terms of PA than netball, there are possibilities to modify variables to be appropriate for netball. By enhancing the PA methods in netball, a more detailed analysis is achieved which will subsequently improve the feedback to coaches and players, and improves coach’s knowledge of PA. By using elite level netball SMEs to develop the model ensures that the novel methods identified here will be usable in practice.

A final point of consideration relates to the lack of sports science research that has applied systems thinking approaches in women’s sports contexts. Thus, this research addresses two important needs. First, netball is an under-researched area relative to other team-based sports contexts, and the use of a systems-based PA framework has been beneficial at identifying non-traditional aspects of on-court performances that require the attention of coaches and players. Secondly, this research should encourage sports scientists to apply other systems thinking approaches to optimize performance in women’s sports contexts – whether this be netball or otherwise. For example, the next step following this research may be to explore how best to measure, test and validate the novel performance components identified and question the feasibility of doing so from a practical and coaching standpoint. This way, the growth of women’s sport can parallel scientific advancement and innovation in the sports sciences, bringing with it the formalization of systems thinking approaches in a underrepresented athletic group.

### Limitations

A potential limitation of the current study is the small number of SMEs used to develop the WDA model of elite level netball. However, the SMEs who participated in the current study have extensive experience in elite level netball including participation in national and international competitions as coaches, analysts, and players. Furthermore, a similar model of football performance used eight SMEs in their analysis (McLean et al., 2017b), and other research using this method has ranged from three to eleven SMEs to develop similar models (Jenkins et al., 2011; Salmon et al., 2016). In addition, the demographics of the SMEs spanned three of the top nations in world netball (Australia, New Zealand, and South Africa). A limitation of the WDA method is that is does not weight the relationships between the nodes to determine nodal importance. However, the purpose of the WDA phase of the CWA framework is to model the entire netball performance system, and in doing so identify all the components within the system that influence the functions and measures of performance. Future research could attempt to weight the relationships between nodes to determine nodal importance.

## Conclusion

The current study provided a first-of-its-kind complex systems model of the elite women’s netball match performance system. The model depicts the key components of netball match performance along with the means-ends relationships between them. It is concluded that, whilst netball performance is complex in nature, there are various opportunities to understand this complexity through the introduction of new PA measures. Further investigation is recommended, particularly the development, testing and validation of the new measures discussed. It is hoped that researchers and practitioners use the model presented in this article to facilitate this, and that netball PA is optimized as a result.

## Author Contributions

SM, AH, GR, and PS conceived the study. SM, AH, GR, AB, and PS performed the study. SM, AH, MM, GR, AB, and PS analyzed the data, wrote and revised the manuscript.

## Conflict of Interest Statement

The authors declare that the research was conducted in the absence of any commercial or financial relationships that could be construed as a potential conflict of interest.

## Appendix 1 | Sme Modifications to the Initial Wda.

**Table 3:**

Level of abstraction	Action taken
Functional purposes	***Minor rewording:*** ‘Play in line with club values’ > ‘demonstrate team values’
	***Added nodes:*** ‘Provide entertainment’
Values and priority measures	***Minor rewording:*** ‘Goals scored/conceded’ > ‘goals scored/missed/conceded’; ‘center restarts’ > ‘center pass conversions’
	***Removed nodes:*** ‘Distance covered’; ‘center restarts’; ‘goal assists’; ‘goal attempt blocks’; ‘number of possessions’
	***Added nodes:*** ‘Psychological flow’; ‘player workload’; ‘penetrative passes’; ‘converted edge feeds’; ‘contested errors/uncontested errors’; ‘time outs’; ‘scoreboard pressure’; ‘passing chains’; ‘defensive/offensive pressure’
	***Nodes incorporated elsewhere:*** ’Handling errors’; ‘intercepts’; ‘loose ball wins’ incorporated into ‘turnovers to score’ ‘Discipline’ incorporated into ‘penalties received/conceded’ ‘Defensive block’; ‘goal attempt blocks’ incorporated into ‘defensive pressure’
Purpose related functions	***Minor rewording:*** ‘Mange tempo’ > ‘control momentum’ ‘Maintain structures’ > ‘maintain unit structures’ ‘Adhere to team culture’ > ‘value team culture’
	***Removed node:*** ‘Gain and maintain possession’
	***Added nodes:*** ‘Spatial awareness’; ‘gain possession’; ‘maintain possession’; ‘optimize cognitive performance’; ‘optimize physical performance’; ‘unpredictability to opponents’
Object related processes	***Minor rewording:*** ‘Team support/hinderance’ > ‘team and player support/hinderance’ ‘Visual communication’ > ‘communicates playing position’ ‘Implement rules’ > ‘enactment of game rules’ ‘Stadium atmosphere’ > ‘controls stadium environment’
Physical objects	***Minor rewording:*** ‘Game plan and tactics’ > ‘game plan’
	***Removed node:*** ‘Air conditioning’
	***Added nodes:*** ‘Benches’; ‘stadium utilities’
	***Nodes incorporated elsewhere:*** ‘GPS’ incorporated into ‘data capturing equipment’

## References

[B1] Bailey J. A. Gastin P. B. Mackey L. Dwyer D. B. (2017). The player load associated with typical activities in elite netball. *Int. J. Sports Physiol. Perform.* 12 1218–1223. 10.1123/ijspp.2016-0378 28182504

[B2] Bakker A. B. Oerlemans W. Demerouti E. Slot B. B. Ali D. K. (2011). Flow and performance: a study among talented Dutch soccer players. *Psychol. Sport Exer.* 12 442–450. 10.1016/j.psychsport.2011.02.003

[B3] Balague N. Torrents C. Hristovski R. Davids K. Araújo D. (2013). Overview of complex systems in sport. *J. Syst. Sci. Complex.* 26 4–13. 10.1007/s11424-013-2285-0

[B4] Bartlett R. Button C. Robins M. Dutt-Mazumder A. Kennedy G. (2012). Analysing team coordination patterns from player movement trajectories in soccer: methodological considerations. *Int. J. Perform. Anal. Sport* 12 398–424. 10.1080/24748668.2012.11868607

[B5] Bishop D. (2008). An applied research model for the sport sciences. *Sports Med.* 38 253–263. 10.2165/00007256-200838030-00005 18278985

[B6] Bishop L. Barnes A. (2013). Performance indicators that discriminate winning and losing in the knockout stages of the 2011 Rugby World Cup. *Int. J. Perform. Anal. Sport* 13 149–159. 10.1080/24748668.2013.11868638

[B7] Bourbousson J. Sève C. McGarry T. (2010). Space–time coordination dynamics in basketball: part 1. Intra-and inter-couplings among player dyads. *J. Sports Sci.* 28 339–347. 10.1080/02640410903503632 20131146

[B8] Bradley P. S. Carling C. Archer D. Roberts J. Dodds A. Di Mascio M. (2011). The effect of playing formation on high-intensity running and technical profiles in English FA Premier League soccer matches. *J. Sports Sci.* 29 821–830. 10.1080/02640414.2011.561868 21512949

[B9] Bruce L. Farrow D. Raynor A. May E. (2009). Notation analysis of skill expertise differences in netball. *Int. J. Perform. Anal. Sport* 9 245–254. 10.1080/24748668.2009.11868481

[B10] Buchheit M. Mendez-Villanueva A. Simpson B. Bourdon P. (2010). Repeated-sprint sequences during youth soccer matches. *Int. J. Sports Med.* 31 709–716. 10.1055/s-0030-1261897 20617485

[B11] Carling C. Wright C. Nelson L. J. Bradley P. S. (2014). Comment on ‘Performance analysis in football: a critical review and implications for future research’. *J. Sports Sci.* 32 2–7. 10.1080/02640414.2013.807352 23886412

[B12] Clemente F. M. Martins F. M. L. Kalamaras D. Wong D. P. Mendes R. S. (2015a). General network analysis of national soccer teams in FIFA World Cup 2014. *Int. J. Perform. Anal. Sport* 15 80–96. 10.1080/24748668.2015.11868778

[B13] Clemente F. M. Martins F. M. L. Wong D. P. Kalamaras D. Mendes R. S. (2015b). Midfielder as the prominent participant in the building attack: a network analysis of national teams in FIFA World Cup 2014. *Int. J. Perform. Anal. Sport* 15 704–722. 10.1080/24748668.2015.11868825

[B14] Cormack S. J. Smith R. L. Mooney M. M. Young W. B. O’Brien B. J. (2014). Accelerometer load as a measure of activity profile in different standards of netball match play. *Int. J. Sports Physiol. Perform.* 9 283–291. 10.1123/ijspp.2012-0216 23799824

[B15] Cornelissen M. McClure R. Salmon P. M. Stanton N. A. (2014). Validating the strategies analysis diagram: assessing the reliability and validity of a formative method. *Appl. Ergon.* 45 1484–1494. 10.1016/j.apergo.2014.04.010 24794935

[B16] Croft H. Willcox B. Lamb P. (2017). Using performance data to identify styles of play in netball: an alternative to performance indicators. *Int. J. Perform. Anal. Sport* 17 1034–1043. 10.1080/24748668.2017.1419408

[B17] Csikszentmihalyi M. (1990). *Flow: The Psychology of Optimal Performance.* New York, NY: Cambridge University Press.

[B18] D’Amour A. Cervone D. Bornn L. Goldsberry K. (2015). *Move or Die: How Ball Movement Creates Open Shots in the NBA.* Boston, MA: MIT Sloan Sports Analytics Conference.

[B19] Davidson A. Trewartha G. (2008). Understanding the physiological demands of netball: a time-motion investigation. *Int. J. Perform. Anal. Sport* 8 1–17. 10.1080/24748668.2008.11868443

[B20] Duarte R. Araújo D. Correia V. Davids K. (2012). Sports teams as superorganisms. *Sports Med.* 42 633–642. 10.2165/11632450-000000000-00000 22715927

[B21] Duarte R. Araújo D. Folgado H. Esteves P. Marques P. Davids K. (2013). Capturing complex, non-linear team behaviours during competitive football performance. *J. Syst. Sci. Complex.* 26 62–72. 10.1007/s11424-013-2290-3

[B22] Eccles D. W. Tenenbaum G. (2004). Why an expert team is more than a team of experts: a social-cognitive conceptualization of team coordination and communication in sport. *J. Sport Exerc. Psychol.* 26 542–560. 10.1123/jsep.26.4.542

[B23] Fish K. Greig M. (2014). The influence of playing position on the biomechanical demands of netball match-play. *J. Athl. Enhanc.* 3 1–5.

[B24] Frencken W. Poel H. D. Visscher C. Lemmink K. (2012). Variability of inter-team distances associated with match events in elite-standard soccer. *J. Sports Sci.* 30 1207–1213. 10.1080/02640414.2012.703783 22788797

[B25] Gómez M.-A. Lorenzo A. Ibañez S.-J. Sampaio J. (2013). Ball possession effectiveness in men’s and women’s elite basketball according to situational variables in different game periods. *J. Sports Sci.* 31 1578–1587. 10.1080/02640414.2013.792942 23679867

[B26] Gómez M. -Á DelaSerna A. Lupo C. Sampaio J. (2014). Effects of situational variables and starting quarter score in the outcome of elite women’s water polo game quarters. *Int. J. Perform. Anal. Sport* 14 73–83. 10.1097/JSC.0000000000000234 26999289

[B27] Gómez M. Á Lorenzo A. Jiménez S. Navarro R. M. Sampaio J. (2015). Examining choking in basketball: effects of game outcome and situational variables during last 5 minutes and overtimes. *Percept. Mot. Skills* 120 111–124. 10.2466/25.29.PMS.120v11x0 25578488

[B28] Gonçalves B. Coutinho D. Santos S. Lago-Penas C. Jiménez S. Sampaio J. (2017). Exploring team passing networks and player movement dynamics in youth association football. *PLoS One* 12:e0171156. 10.1371/journal.pone.0171156 28141823 PMC5283742

[B29] Higham D. G. Hopkins W. G. Pyne D. B. Anson J. M. (2014). Performance indicators related to points scoring and winning in international rugby sevens. *J. Sports Sci. Med.* 13 358–364. 24790490 PMC3990890

[B30] Hill D. M. Hanton S. Fleming S. Matthews N. (2009). A re-examination of choking in sport. *Eur. J. Sport Sci.* 9 203–212. 10.1080/17461390902818278

[B31] Hill D. M. Hanton S. Matthews N. Fleming S. (2010). Choking in sport: a review. *Int. Rev. Sport Exerc. Psychol.* 3 24–39. 10.1080/17509840903301199

[B32] Hobbs W. Morgan S. Gorman A. D. Mooney M. Freeston J. (2018). Playing unpredictably: measuring the entropy of ball trajectories in international women’s basketball. *Int. J. Perform. Anal. Sport* 18 1–12. 10.1080/24748668.2018.1453639

[B33] Hopper D. M. Hopper J. L. Elliott B. C. (1995). Do selected kinanthropometric and performance variables predict injuries in female netball players? *J. Sports Sci.* 13 213–222. 10.1080/02640419508732230 7563288

[B34] Hughes M. Franks I. M. (2004). *Notational Analysis of Sport: Systems for Better Coaching and Performance in Sport.* London: Psychology Press.

[B35] Hughes M. D. Bartlett R. M. (2002). The use of performance indicators in performance analysis. *J. Sports Sci.* 20 739–754. 10.1080/026404102320675602 12363292

[B36] Hulme A. Salmon P. Nielsen R. Read G. J. Finch C. (2017). Closing Pandora’s box: adapting a systems ergonomics methodology for better understanding the ecological complexity underpinning the development and prevention of running-related injury. *Theor. Issues Ergon. Sci.* 18 338–359. 10.1080/1463922X.2016.1274455

[B37] Hulme A. Thompson J. Plant K. L. Read G. J. Mclean S. Clacy A. (2018). Applying systems ergonomics methods in sport: a systematic review. *Appl. Ergon.* 10.1016/j.apergo.2018.03.019 [Epub ahead of print]. 29674008

[B38] Hume P. A. Steele J. R. (2000). A preliminary investigation of injury prevention strategies in Netball: are players heeding the advice? *Journal of Science and Medicine in Sport* 3 406–413. 11235006 10.1016/s1440-2440(00)80007-9

[B39] Jackson S. A. Marsh H. W. (1996). Development and validation of a scale to measure optimal experience: the flow state scale. *J. Sport Exerc. Psychol.* 18 17–35. 10.1123/jsep.18.1.17

[B40] Jenkins D. P. Stanton N. A. Salmon P. M. Walker G. H. (2011). Using work domain analysis to evaluate the impact of technological change on the performance of complex socio-technical systems. *Theor. Issues Ergon. Sci.* 12 1–14. 10.1080/14639220903353401

[B41] Lago-Peñas C. Gómez A. M. Viaño J. González-García I. Fernández-Villarino M. d. L. á (2013). Home advantage in elite handball: the impact of the quality of opposition on team performance. *Int. J. Perform. Anal. Sport* 13 724–733. 10.1080/24748668.2013.11868684

[B42] Lupo C. Condello G. Capranica L. Tessitore A. (2014). Women’s water polo World Championships: technical and tactical aspects of winning and losing teams in close and unbalanced games. *J. Strength Cond. Res.* 28 210–222. 10.1519/JSC.0b013e3182955d90 23588481

[B43] Mackenzie R. Cushion C. (2013). Performance analysis in football: a critical review and implications for future research. *J. Sports Sci.* 31 639–676. 10.1080/02640414.2012.746720 23249092

[B44] Mason-Mackay A. R. Whatman C. Reid D. Lorimer A. (2016). The effect of ankle bracing on landing biomechanics in female netballers. *Phys. Ther. Sport* 20 13–18. 10.1016/j.ptsp.2015.11.002 27325534

[B45] Mclean S. Salmon P. (2019). The weakest link: a novel use of network analysis for the broken passing links in football. *Sci. Med. Football* 1–4. 10.1080/24733938.2018.1562277

[B46] McLean S. Salmon P. Gorman A. Dodd K. Solomon C. (2018a). Integrating communication and passing networks in football using social network analysis. *Sci. Med. Football.* 10.1080/24733938.2018.1478122

[B47] McLean S. Salmon P. Gorman A. Wickham J. Berber E. Solomon C. (2018b). The effect of playing formation the passing network characteristics of a professional football team. *Hum. Mov.* 19 14–22. 10.5114/hm.2018.79416

[B48] McLean S. Salmon P. M. Gorman A. D. Naughton M. Solomon C. (2017a). Do inter-continental playing styles exist? Using social network analysis to compare goals from the 2016 EURO and COPA football tournaments knock-out stages. *Theor. Issues Ergon. Sci.* 18 370–383. 10.1080/1463922X.2017.1290158

[B49] McLean S. Salmon P. M. Gorman A. D. Read G. J. Solomon C. (2017b). What’s in a game? A systems approach to enhancing performance analysis in football. *PLoS One* 12:e0172565. 10.1371/journal.pone.0172565 28212392 PMC5315401

[B50] McLean S. Salmon P. M. Gorman A. D. Stevens N. J. Solomon C. (2017c). A social network analysis of the goal scoring passing networks of the 2016 European Football Championships. *Hum. Mov. Sci.* 57 400–408. 10.1016/j.humov.2017.10.001 29046222

[B51] McManus A. Stevenson M. R. Finch C. F. (2006). Incidence and risk factors for injury in non-elite netball. *J. Sci. Med. Sport* 9 119–124. 10.1016/j.jsams.2006.03.005 16621712

[B52] Meletakos P. Vagenas G. Bayios I. (2011). A multivariate assessment of offensive performance indicators in men’s handball: trends and differences in the World Championships. *Int. J. Perform. Anal. Sport* 11 284–294. 10.1080/24748668.2011.11868548

[B53] Moura F. A. Martins L. E. B. Anido R. D. O. De Barros R. M. L. Cunha S. A. (2012). Quantitative analysis of Brazilian football players’ organisation on the pitch. *Sports Biomech.* 11 85–96. 10.1080/14763141.2011.637123 22518947

[B54] Naikar N. (2013). *Work Domain Analysis: Concepts, Guidelines, and Cases.* Boca Raton, FL: CRC Press 10.1201/b14774

[B55] Naikar N. Sanderson P. M. Lintern G. (1999). Work domain analysis for identification of training needs and training-system design. *Paper Presented at the Proceedings of the Human Factors and Ergonomics Society Annual Meeting* Washington, DC 10.1177/154193129904302103

[B56] Netball Australia (2018). *History of Netball.* Available at: http://netball.com.au/about-netball-australia/history-of-netball/ [acessed October 19 2018].

[B57] O’Donoghue P. Mayes A. Edwards K. M. Garland J. (2008). Performance norms for British national super league netball. *Int. J. Sports Sci. Coach.* 3 501–511. 10.1260/174795408787186486

[B58] Oliveira T. Gómez M. Sampaio J. (2012). Effects of game location, period, and quality of opposition in elite handball performances. *Percept. Mot. Skills* 114 783–794. 10.2466/30.06.PMS.114.3.783-794 22913020

[B59] Pulling C. Eldridge D. Lomax J. (2016). Centre passes in the UK netball super league. *Int. J. Perform. Anal. Sport* 16 389–400. 10.1080/24748668.2016.11868894

[B60] Read G. J. Salmon P. M. Lenné M. G. Stanton N. A. (2015). Designing sociotechnical systems with cognitive work analysis: putting theory back into practice. *Ergonomics* 58 822–851. 10.1080/00140139.2014.980335 25407778

[B61] Read G. J. Salmon P. M. Lenné M. G. Stanton N. A. (2016). Walking the line: understanding pedestrian behaviour and risk at rail level crossings with cognitive work analysis. *Appl. Ergon.* 53 209–227. 10.1016/j.apergo.2015.10.004 26518501

[B62] Rein R. Memmert D. (2016). Big data and tactical analysis in elite soccer: future challenges and opportunities for sports science. *Springerplus* 5:1410. 10.1186/s40064-016-3108-2 27610328 PMC4996805

[B63] Rein R. Raabe D. Memmert D. (2017). Which pass is better?” Novel approaches to assess passing effectiveness in elite soccer. *Hum. Mov. Sci.* 55 172–181. 10.1016/j.humov.2017.07.010 28837900

[B64] Ribeiro J. Silva P. Duarte R. Davids K. Garganta J. (2017). Team sports performance analysed through the lens of social network theory: implications for research and practice. *Sports Med.* 47 1689–1696. 10.1007/s40279-017-0695-1 28197801

[B65] Robertson S. Back N. Bartlett J. D. (2016). Explaining match outcome in elite Australian rules football using team performance indicators. *J. Sports Sci.* 34 637–644. 10.1080/02640414.2015.1066026 26176890

[B66] Robertson S. Woods C. Gastin P. (2015). Predicting higher selection in elite junior Australian rules football: the influence of physical performance and anthropometric attributes. *J. Sci. Med. Sport* 18 601–606. 10.1016/j.jsams.2014.07.019 25154704

[B67] Ruano M. Á Serna A. D. Lupo C. Sampaio J. E. (2016). Effects of game location, quality of opposition, and starting quarter score in the outcome of elite water polo quarters. *J. Strength Cond. Res.* 30 1014–1020. 10.1097/JSC.0000000000000234 26999289

[B68] Salmon P. M. Clacy A. Dallat C. (2017a). It’s not all about the bike: distributed situation awareness and teamwork in elite women’s cycling teams. *Contemp. Ergon.* 2017 240–248.

[B69] Salmon P. M. Walker G. H. Stanton N. A. Jenkins D. P. (2017b). *Cognitive Work Analysis: Applications, Extensions and Future Directions.* Boca Raton, FL: CRC Press.

[B70] Salmon P. M. Lenné M. G. Read G. J. Mulvihill C. M. Cornelissen M. Walker G. (2016). More than meets the eye: using cognitive work analysis to identify design requirements for future rail level crossing systems. *Appl. Ergon.* 53 312–322. 10.1016/j.apergo.2015.06.021 26143077

[B71] Sampaio J. Maçãs V. (2012). Measuring tactical behaviour in football. *Int. J. Sports Med.* 33 395–401. 10.1055/s-0031-1301320 22377947

[B72] Sampaio J. McGarry T. Calleja-González J. Sáiz S. J. del Alcázar X. S. Balciunas M. (2015). Exploring game performance in the National basketball association using player tracking data. *PLoS One* 10:e0132894. 10.1371/journal.pone.0132894 26171606 PMC4501835

[B73] Sarmento H. Clemente F. M. Araújo D. Davids K. McRobert A. Figueiredo A. (2017). What performance analysts need to know about research trends in Association football (2012–2016): a systematic review. *Sports Med.* 48 799–836. 10.1007/s40279-017-0836-6 29243038

[B74] Sarmento H. Marcelino R. Anguera M. T. CampaniÇo J. Matos N. LeitÃo J. C. (2014). Match analysis in football: a systematic review. *J. Sports Sci.* 32 1831–1843. 10.1080/02640414.2014.898852 24787442

[B75] Sinclair J. Chockalingam N. Naemi R. Vincent H. (2015). The effects of sport-specific and minimalist footwear on the kinetics and kinematics of three netball-specific movements. *Footwear Sci.* 7 31–36. 10.1080/19424280.2014.983445

[B76] Stanton N. A. Bessell K. (2014). How a submarine returns to periscope depth: analysing complex socio-technical systems using cognitive work analysis. *Appl. Ergon.* 45 110–125. 10.1016/j.apergo.2013.04.022 23702259

[B77] Swann C. Keegan R. J. Piggott D. Crust L. (2012). A systematic review of the experience, occurrence, and controllability of flow states in elite sport. *Psychol. Sport Exerc.* 13 807–819. 10.1016/j.psychsport.2012.05.006

[B78] Tenga A. Kanstad D. Ronglan L. Bahr R. (2009). Developing a new method for team match performance analysis in professional soccer and testing its reliability. *Int. J. Perform. Anal. Sport* 9 8–25. 10.1080/24748668.2009.11868461

[B79] Tenga A. Mortensholm A. O’Donoghue P. (2017). Opposition interaction in creating penetration during match play in elite soccer: evidence from UEFA champions league matches. *Int. J. Perform. Anal. Sport* 17 802–812. 10.1080/24748668.2017.1399326

[B80] Thomas C. Ismail K. T. Simpson R. Comfort P. Jones P. A. Dos’Santos T. (2017). Physical profiles of female academy netball players by position. *J. Strength Cond. Res.* 10.1519/JSC.0000000000001949 [Epub ahead of print]. 28426516

[B81] Travassos B. Davids K. Araújo D. Esteves P. T. (2013). Performance analysis in team sports: advances from an ecological dynamics approach. *Int. J. Perform. Anal. Sport* 13 83–95. 10.1080/24748668.2013.11868633

[B82] Turner V. Turner V. W. Victor T. (1967). *The Forest of Symbols: Aspects of Ndembu Ritual* Vol. 101 New York, NY: Cornell University Press

[B83] Vaz L. Van Rooyen M. Sampaio J. (2010). Rugby game-related statistics that discriminate between winning and losing teams in IRB and Super twelve close games. *J. Sports Sci. Med.* 9:51. 24149385 PMC3737978

[B84] Vicente K. J. (1999). *Cognitive Work Analysis: Toward Safe, Productive, and Healthy Computer-Based Work.* Boca Raton, FL: CRC Press.

[B85] Villarejo D. Ortega E. Gómez M. -Á Palao J.-M. (2014). Design, validation, and reliability of an observational instrument for ball possessions in rugby union. *Int. J. Perform. Anal. Sport* 14 955–967. 10.1080/24748668.2014.11868771

[B86] Wallace J. L. Norton K. I. (2014). Evolution of World cup soccer final games 1966–2010: game structure, speed and play patterns. *J. Sci. Med. Sport* 17 223–228. 10.1016/j.jsams.2013.03.016 23643671

[B87] Wäsche H. Dickson G. Woll A. Brandes U. (2017). Social network analysis in sport research: an emerging paradigm. *Eur. J. Sport Soc.* 14 138–165. 10.1080/16138171.2017.1318198

